# Choosing Kinase Inhibitors for Androgen Deprivation Therapy-Resistant Prostate Cancer

**DOI:** 10.3390/pharmaceutics14030498

**Published:** 2022-02-24

**Authors:** Shangwei Zhong, Shoujiao Peng, Zihua Chen, Zhikang Chen, Jun-Li Luo

**Affiliations:** 1 Department of General Surgery, Xiangya Hospital, Central South University, Hunan 410008, China; swzhong@csu.edu.cn (S.Z.); pengshj08@163.com (S.P.); zihuachenxy@126.com (Z.C.); 2 Department of Molecular Medicine, The Scripps Research Institute, Jupiter, FL 33459, USA

**Keywords:** androgen deprivation therapy (ADT), castration-resistant prostate cancer (CRPC), kinase, kinase inhibitors, IκB kinase (IKK), TANK-binding kinase 1 (TBK1), PIM1 kinase, tumor progression locus 2 (Tpl2), NEK6 (a mitotic-related serine/threonine kinase), glycogen-synthase-kinase 3β (GSK-3β), T-LAK cell-originated protein kinase (TOPK), cyclin-dependent kinases (CDKs), bromodomain (BRD)-containing kinases (BETs), aurora kinase A (AURKA), AMP-activated protein kinase (AMPK), mitogen-activated protein kinase (MAPK), jun kinase (JNK), phosphatidylinositol-3-kinase (PI3K)/AKT, polo-like kinase 1 (PLK1), LIM-domain kinase-2 (LIMK2), the receptor tyrosine kinases (RTKs), the non-receptor tyrosine kinases (NRTKs), phosphatidylinositol 4-phosphate 5-kinase type 1α (PIP5K1α), sphingosine kinases (SKs), hexokinase, phosphofructokinase (PFK), riboflavin kinases (RFK), thymidine kinase, protein kinase A (PKA)

## Abstract

Androgen deprivation therapy (ADT) is a systemic therapy for advanced prostate cancer (PCa). Although most patients initially respond to ADT, almost all cancers eventually develop castration resistance. Castration-resistant PCa (CRPC) is associated with a very poor prognosis, and the treatment of which is a serious clinical challenge. Accumulating evidence suggests that abnormal expression and activation of various kinases are associated with the emergence and maintenance of CRPC. Many efforts have been made to develop small molecule inhibitors to target the key kinases in CRPC. These inhibitors are designed to suppress the kinase activity or interrupt kinase-mediated signal pathways that are associated with PCa androgen-independent (AI) growth and CRPC development. In this review, we briefly summarize the roles of the kinases that are abnormally expressed and/or activated in CRPC and the recent advances in the development of small molecule inhibitors that target kinases for the treatment of CRPC.

## 1. Introduction

Kinases are part of the large family of phosphotransferase, which catalyze the transfer of a phosphate moiety from a high energy molecule (such as ATP) to their substrate molecule. More than five hundred different kinases have been identified in humans, the majority of which phosphorylate proteins while some kinases act on small molecules such as lipids, carbohydrates, amino acids, and nucleotides. For example, androgen receptor (AR) is a substrate of PIM1 and activated CDC42-associated kinase-1 (ACK1), lipid is the substrate of phosphatidylinositol 4-phosphate 5-kinase type 1α (PIP5K1α), and fructose-6-phosphate is one of the substrates of phosphofructokinase (PFK). Based on their substrates, kinases are classified into protein kinases, lipid kinases, and carbohydrate kinases [1]. Protein kinases phosphorylate the amino acid serine, threonine, tyrosine, or histidine residues of the proteins. A specific protein kinase, such as IκB kinase and TANK-binding Kinase 1 (TBK1), normally has more than one substrate, and a specific protein, such as twist-related protein 1 (TWIST1), can serve as a substrate for multiple kinases. Kinases are fundamental molecules in both normal and cancer cells, and are involved in almost all cellular activities [2]. The kinases-mediated protein post-translational modifications function as key mediators or switches in many critical cellular signal pathways. For instance, NEK6 interacts with STAT3 and induces STAT3-mediated transcriptional activation through phosphorylating STAT3 [3]. Kinase abnormal activation and dysregulation have been associated with many diseases, such as autoimmune diseases and cancer.

Prostate cancer (PCa) is the most common malignancy, and the second-leading cause of cancer-related mortality in men in Western countries [4]. In tumors confined to the prostate, radical prostatectomy and radiotherapy are effective; however, for late stage disseminated disease, current therapies are merely palliative. Androgen deprivation therapy (ADT) is a systemic therapy for advanced PCa. Although a majority of patients initially respond to ADT, the responses are transient and almost all cancers eventually develop castration resistance [4,5,6,7,8,9]. Castration-resistant PCa (CRPC) is associated with a very poor prognosis, and the treatment of which is a serious clinical challenge [4,5,6,7]. Abnormal expression and activation of many kinases have been associated with PCa development, progression, as well as the emergence and maintenance of CRPC. Recently, many kinases, such as polo-like kinase (PLK1), aurora kinase A (AURKA), glycogen-synthase-kinase 3 (GSK-3), and PIP5K1α have been identified to participate in CRPC progression through regulating their substrates and/or downstream signal pathways, targeting these kinases with inhibitors can significantly interrupt the pathogenesis of CRPC (Table 1). For instance, LIM-domain kinase-2 (LIMK2) is up-regulated after ADT and promotes CRPC development through phosphorylating TWIST1 in CRPC cells. Knockdown of LIMK2 significantly suppresses CRPC development [10]. As many kinases play important roles in CRPC growth and development, kinase targeted therapies have been developed for the treatment of CRPC.

**Figure 1:**
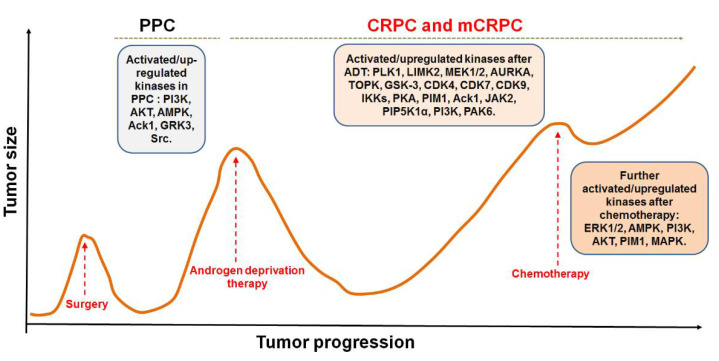
Diagram of prostate cancer progression and the activated/up-regulated kinases in primary prostate cancer (PPC), castration-resistant prostate cancer (CRPC), and metastatic CRPC (mCRPC).

**Figure 2:**
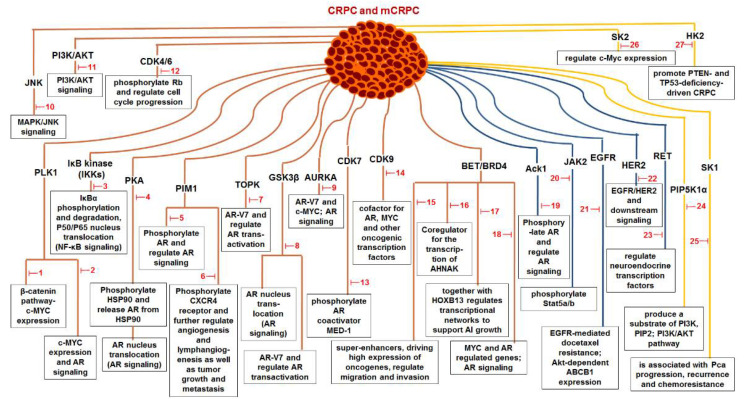
Therapeutic strategies that target key kinases in CRPC. Note: 1: combined GSK461364A with JQ1; 2: WNY0824; 3: ursolic acid/apigenin/α-tomatine; 4: H89; 5: compound 9; 6: DHPCC-9; 7: OTS-514; 8: LY-2090314; 9: S1451/alisertib(MLN8237); 10: AS602801; 11: combined wortmannin with MK2206; 12: pso; 13: THZ1; 14: KB-0742; 15: JQ1; 16: MZ1; 17: MA4-022-1; 18: 6i/7m/(R)-12/ABBV-075/compound 7d; 19: AIM-100; 20: β-elemonic acid; 21: Gefitinib; 22: dacomitinib; 23: AD80; 24: ISA-2011B; 25: FTY720; 26: ABC294640; 27: 2-deoxyglucose.

Small molecule inhibitors are attractive anti-tumor agents due to their small size, well-penetrative features, and the potential to be engineered for oral administration [11,12]. Recently, many small molecule inhibitors have been developed to specifically or mainly target the key kinases involved in CRPC emergence and maintenance, some of them have been approved by U.S. Food and Drug Administration (FDA) [11,12]. These small molecules are designed to inhibit kinase activity or interrupt kinase-mediated signal pathways that are associated with PCa androgen-independent (AI) growth, progression, and metastasis (Figure 1 and Figure 2, Table 1). Notably, some of these kinase inhibitors have been applied in clinical trials (Table 2). Here, we briefly review the roles of the key kinases in CRPC development and summarize the recent advances in the development of small molecule inhibitors that target kinases for the treatment of CRPC.

**Table 1:** Inhibitors target key kinases in CRPC.

Kinase	Role in CRPC	Inhibitor	Chemical Structure	Mechanism of Inhibition	Reference
PLK1	PLK1 signaling is one of the most up-regulated pathways after androgen deprive in mouse xenograft. PLK1 activation promotes CRPC development through affecting the PI3K–AKT–mTOR pathway and androgen receptor (AR) signaling.	BI2536 (combined with olaparib)	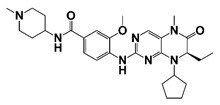	Since monotherapy with olaparib leads to high expression of PLK1, PLK1 inhibitor can significantly increase the efficacy of olaparib.	[13]
		GSK461364A (combined with BRD4 inhibitor, JQ1)	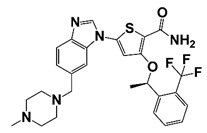	Inhibits PLK1, c-MYC expression and AR signaling	[14]
		WNY0824	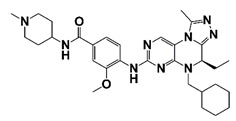	Inhibit AR transcriptional program, down-regulate MYC and induce mitotic abnormality	[15]
BRD4/BET	Bind to SEs that drive high expression of oncogenes; BRD4 together with HOXB13 regulated transcriptional networks to support androgen-independent cell proliferation of CRPC	JQ1 (combined with PLK1 inhibitor, GSK461364A)	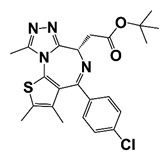	Inhibits PLK1, c-MYC expression and AR signaling; down-regulating SE-associated genes, including genes involved in migration and invasion, especially a long non-coding RNA, MANCR	[14,16]
		MA4-022-1 compound	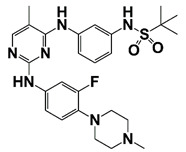	Interfere BRD4-HOXB13-HOTBIN10 regulatory circuit	[17]
		(R)-12 (Y02234)	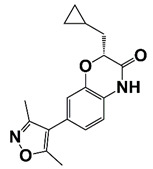	Bind to BRD4 and block the interaction between bromodomain and acetyl lysine; suppress ERG, Myc, and AR signaling	[18]
		6i (Y06036) and 7m (Y06137)	6i 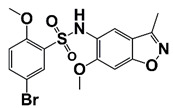	Bind to BRD4 and suppress expression of MYC and AR regulated genes	[19]
			7m 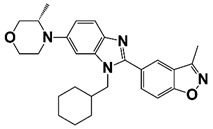		
		ABBV-075	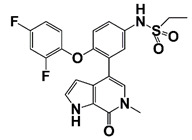	Disrupt the recruitment of BRD4 to gene-regulatory regions co-occupied by AR, including PSA and TMPRSS2 enhancers, leading to the inhibition of transcription of AR target genes	[20]
		Compound 7d	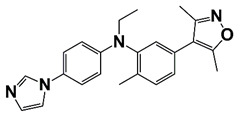	Disrupt the functions of AR and BET via binding to AR and suppressing transactivation of AR as well as AR mutant	[21]
LIMK2	LIMK2 involves in CRPC progression through increasing TWIST1 mRNA levels and stabilizing TWIST1 by phosphorylation, mediating SPOP degradation	LIMK2 allosteric inhibitor (sulfonamide 2) (combined with docetaxel)	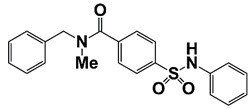	Is non-ATP competitive (allosteric inhibition) and binds in the DFG-out orientation of kinase	[10,22]
MEK	MEK1/2 promotes CRPC progression via regulating their downstream target such as ERK1	Selumetinib (AZD6244) (combined with ricolinostat (ACY1215))	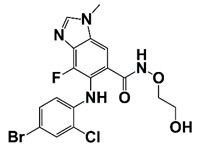	Decrease AR-dependent gene (KLK2, DUSP1) mRNA levels; increase AR cytoplasmatic expression	[23,24]
AURKA	AURKA is overexpressed in CRPC, regulates androgen receptor variants (AR-Vs) expression, and acts as a phosphatase and functions through regulating its substrates, including YBX1, TWIST1, and LIMK2.	MLN8237 (alisertib)	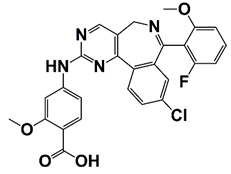	Inhibit the growth of CRPC cells with high levels of AR	[7]
		S1451 (TC-S 7010)	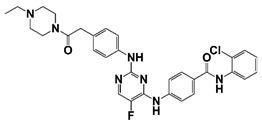	Decrease AR-V7 levels and markedly reduce AR-V-driven proliferation and survival of CRPC cells	[25]
TOPK	TOPK drives androgen-independent growth in prostate cancer cells (LNCaP and VCaP) via enhancing androgen receptor splice variant (ARv7)	OTS-514	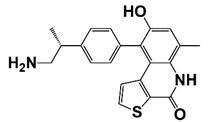	Repress AR transactivation, and AR stability	[26]
GSK-3	Promote AR-V7 transcription	LY 2090314	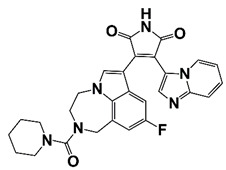	Activate β-catenin signaling, which suppressed AR-V7 transcriptional activity	[27]
CDK9	A cofactor for AR, MYC and other oncogenic transcription factors	KB-0742	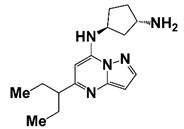	Down-regulate nascent transcription, especially AR-driven oncogenic programs and short half-life transcripts	[28]
CDK4	Regulate cell cycle progression	Pso (3, 9-dihydroxy-2-prenylco- umestan (psoralidin))	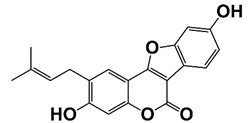	Induce G0/G1 cell cycle arrest and cell growth inhibition in CRPC cells	[29]
CDK7	Regulate MED1-mediated, AR-dependent oncogenic transcriptional amplification	THZ1	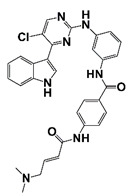	Interfere the recruitment of MED1 to chromatin and further suppress AR target gene expression	[30,31]
IκB kinase (IKK)	Phosphorylate IκBα, resulting in the degradation of IκBα and release of p50/p65. The p50/p65 translocates into nucleus to start the transcription of NF-κB-regulating genes	Ursolic acid	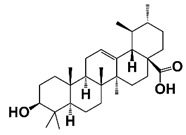	Inhibit IKK activation and phosphorylation of IκBα	[32]
		Apigenin	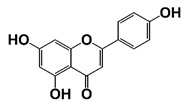	Inhibit IKKα kinase activity, and IκBα phosphorylation and degradation	[33]
		α-tomatine	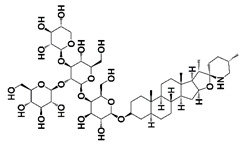	Inhibit IκB kinase-mediated IκBα phosphorylation, IκBα degradation and p50/p65 nuclear translocation	[34]
PKA	PKA activation phosphorylates heat shock protein (HSP90), which binds to the unliganded AR in cytoplasm, and release AR from HSP90. The free AR binds to HSP27 and sequentially migrates into the nucleus	H89	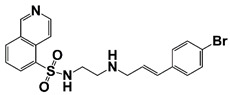	Inhibit the PKA activity and AR translocation	[35]
PIM1	Modulate AR stability and transcriptional activity through phosphorylating AR	Compound 9 (DHPCC-9) and its pyrrolo[2,3-a]carbazole derivatives	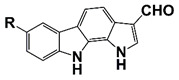 (compound 9 R=H; compound 28 R=Br)	Inhibit the PIM1 kinase activity	[36]
		DHPCC-9 (1,10-dihydropyrrolo[2,3-a]carbazole-3-carbaldehyde)	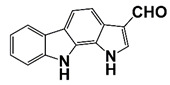	Inhibit angiogenesis and lymphangiogenesis as well as phosphorylation of the CXCR4 chemokine receptor	[37]
Ack1	Phosphorylate AR-tyrosine 267	AIM-100 (4-amino-5,6-biaryl-furo[2,3-d]pyrimidine)	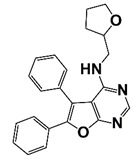	Inhibit Ack1 tyrosine kinase activity and suppress Ack1 mediated ATM	[38]
JAK2	Phosphorylate Stat5a/b, regulate its dimerization, nuclear translocation, DNA binding and transcriptional activity	β-elemonic acid (β-EA)	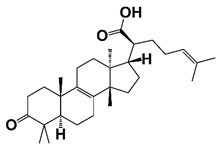	Suppress of JAK2/STAT3/MCL-1 and NF-ĸB signaling pathway	[39]
EGFR	Promote CRPC progression through regulating its downstream signaling; mediated docetaxel resistance in human CRPC via Akt-dependent expression of ABCB1	Gefitinib	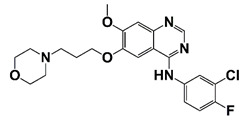	Inhibit EGFR activity	[40]
HER2	Form EGFR/HER2 heterodimerization and regulate downstream signaling to promote CRPC	dacomitinib	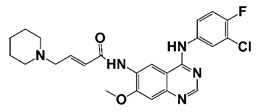	Decrease HER2 protein stability and prevent EGFR/HER2 heterodimerization, therefore interrupting downstream signaling and increasing CRPC cell apoptosis	[41]
RET	Is up-regulated in NEPC, and RET expression correlated with neuroendocrine transcription factors, including POU3F2, SOX2, ONECUT2 and ASCL1	AD80	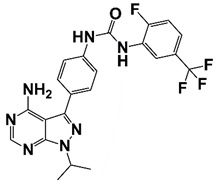	block RET signaling	[42]
PIP5K1α	PIP5K1α produces a substrate of PI3K, PIP2, which is required for the activation of PI3K/AKT pathway	ISA-2011B	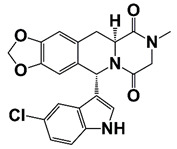	Inhibit AKT activity, reduce invasion, increase cancer cell apoptosis and inhibit tumor growth in CRPC model	[43]
SK1	High levels of SK1 have been identified, and SK1 level and activity are associated with prostate cancer progression, recurrence and chemoresistance	FTY720 (fingolimod)	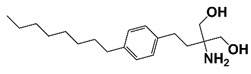	Activate caspase-3 and induce apoptosis	[44,45,46]
SK2	Regulate expression of the oncogene c-Myc and promote PCa progression	ABC294640	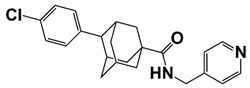	Reduce CRPC cell growth and the expression of c-Myc and AR; inhibit dihydroceramide desaturase (DEGS), resulting in the increase of dihydroceramides	[47]
HK2	Increased HK2 expression and activity is associated with CRPC development, especially PTEN- and TP53-deficiency-driven CRPC	2-deoxyglucose (2-DG)	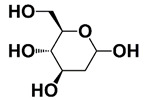	Cause AMPK phosphorylation, leading to inhibit mTORC1-S6K1 translation signaling and sequentially block anti-apoptotic protein MCL-l synthesis	[48]

**Table 2:** Kinase inhibitors for CRPC in clinical trials.

Inhibitor	Chemical Structure	Target	Clinical Trial Phase	Model System/Patient Characteristics	Treatment	Results	Reference
Alisertib	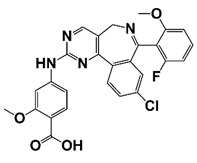	AURKA	Phase II (NCT01799278)	Patients with castration-resistant and neuroendocrine prostate cancer	Alisertib	Some but not all patients with NEPC with Aurora-A and N-Myc activation do benefit from alisertib.	[49]
Alisertib		AURKA	I/II (NCT01848067)	Patients with metastatic castration-resistant prostate cancer	Alisertib in combination with abiraterone and prednisone	No clear signal indicates that alisertib might be beneficial for patients with mCRPC progressing on abiraterone	[50]
Alisertib		AURKA	I (NCT01094288)	Patients with solid tumors, including CRPC	Alisertib combines with docetaxel	1 complete response in a patient with bladder cancer, six partial responses in patients with castration-resistant prostate cancer, and 1 partial response in a patient with angiosarcoma.	[51]
AZD5363 (Capivasertib)	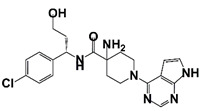	AKT	I (NCT02121639)	Patients with metastatic castration-resistant prostate cancer	AZD5363 combines with docetaxel and prednisolone chemotherapy	PSA reduction to <50% at 12 weeks occurred in seven patients	[52]
AZD5363 (Capivasertib)		AKT	I (NCT02525068)	mCRPC patients who previously failed abiraterone and/or enzalutamide	AZD5363 combines with enzalutamide	Decrease plasma exposure of capivasertib; Three patients show the criteria for response	[53]
BKM120 (Buparlisib)	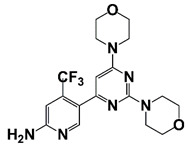	PI3K	II (NCT02035124)	Patients with metastatic castration-resistant prostate cancer	cabazitaxel plus BKM120	Withdrawn due to slow accrual and no patients being enrolled	https://clinicaltrials.gov/ct2/show/NCT02035124?term=PI3K+inhibitor&cond=CRPC&draw=2&rank=1 (30 October 2021)
AZD8186	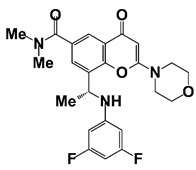	PI3Kbeta	I (NCT03218826)	Patients with advanced solid tumors, including advanced prostate carcinoma	AZD8186 plus docetaxel	Expected completion: 1 April 2022. Outcome measure: Maximum tolerated dose (MTD) and recommended phase 2 dose (RP2D); incidence of adverse events	https://clinicaltrials.gov/ct2/show/NCT03218826?term=PI3K+inhibitor&cond=CRPC&draw=2&rank=3 (30 October 2021)
LY3023414 (Samotolisib)	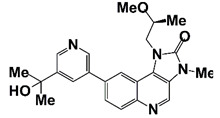	PI3K/mTOR	II (NCT02407054)	Patients with prostate cancer, including metastatic castration-resistant prostate cancer	Enzalutamide plus LY3023414	Outcome measure: progression free survival (PFS); time to disease progression (TTP)	https://clinicaltrials.gov/ct2/show/NCT02407054?term=PI3K+inhibitor&cond=CRPC&draw=2&rank=4 (30 October 2021)
ZEN003694	This compound is under clinical trial and its structure is unpublished.	BET Bromodomain	II (NCT04471974)	Patients with metastatic castration-resistant prostate cancer	ZEN003694 plus enzalutamide and pembrolizumab	Expected completion: 31 December 2025. Outcome measure: composite response rate; progression-free survival (PFS); PSA50 response proportion	https://clinicaltrials.gov/ct2/show/NCT04471974?term=BET+inhibitor&cond=CRPC&draw=2&rank=1 (30 October 2021)
ZEN003694		BET Bromodomain	Ib/IIa (NCT02711956)	Patients with metastatic castration-resistant prostate cancer	ZEN003694 in combination with enzalutamide	Outcome measure: dose escalation and dose confirmation; PSA response rate	https://clinicaltrials.gov/ct2/show/NCT02711956?term=BET+inhibitor&cond=CRPC&draw=2&rank=3 (30 October 2021)
Birabresib (MK-8628/OTX015)	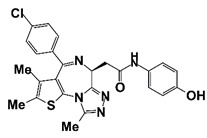	BET Bromodomain	Ib (NCT02259114)	Patients with advanced solid tumors, including castration-resistant prostate carcinoma	Birabresib	Has dose-proportional exposure and a favorable safety profile; recommended phase II with a dose of 80 mg once daily with continuous dosing	[54]
Palbociclib	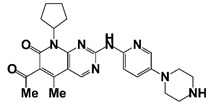	CDK4/6	II (NCT02905318)	Patients with metastatic castration-resistant prostate cancer	Palbociclib	Expected completion: 21 May 2022. Outcome measure: clinical benefit rate; PSA decline ≥ 50%	https://clinicaltrials.gov/ct2/show/NCT02905318?term=CDK+inhibitor&cond=CRPC&draw=2&rank=3 (30 October 2021)
Trametinib	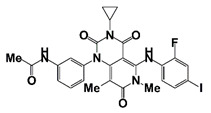	MEK1/2	II (NCT02881242)	Patients with metastatic castration-resistant prostate cancer	Trametinib	Expected completion: 31 January 2023. Outcome measure: PSA response rate; response rate assessed by RECIST criteria; overall survival.	https://clinicaltrials.gov/ct2/show/NCT02881242 (30 October 2021)
CEP-11981 (ESK981)	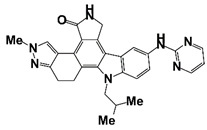	Tyrosine kinase	II (NCT04159896)	Patients with metastatic castration-resistant prostate cancer	ESK981 plus nivolumab	Expected completion: 1 March 2022. Outcome measure: the prostate specific antigen (PSA) >= 50% response rate; the safety and tolerability of ESK981 plus nivolumab	https://clinicaltrials.gov/ct2/show/NCT04159896?term=Tyrosine+kinase+inhibitor&cond=CRPC&draw=2&rank=1 (30 October 2021)
CEP-11981 (ESK981)		Tyrosine kinase	II (NCT03456804)	Patients with metastatic castration-resistant prostate cancer	ESK981	Expected completion: 31 October 2022. Outcome measure: PSA decline of >=50% (PSA50); PSA progression free survival (PFS); Time to PSA response	https://clinicaltrials.gov/ct2/show/NCT03456804?term=Tyrosine+kinase+inhibitor&cond=CRPC&draw=2&rank=2 (30 October 2021)
masitinib	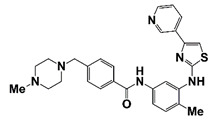	Tyrosine kinase	III (NCT03761225)	Patients with metastatic castration-resistant prostate cancer	Masitinib plus docetaxel	Outcome measure: progression free survival (PFS); overall survival (OS)	https://clinicaltrials.gov/ct2/show/NCT03761225?term=Tyrosine+kinase+inhibitor&cond=CRPC&draw=2&rank=3 (30 October 2021)
Cabozantinib (XL184)	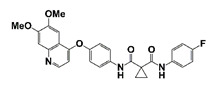	Tyrosine kinase	I (NCT01683994)	Patients with metastatic castration-resistant prostate cancer	Cabozantinib plus docetaxel and prednisone	The median time to progression and overall survival time were 13.6 and 16.3 months; cabozantinib is safely added to docetaxel/ prednisone with possible enhanced efficacy	[55]
lapatinib	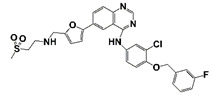	HER2	II (NCT00246753)	Patients with castration-resistant prostate cancer	lapatinib	Show single agent activity in CRPC patients as evaluated by PSA	[56]

## 2. Serine/Threonine Kinase and Their Therapeutic Implications

### 2.1. IκB Kinases (IKKs)

NF-κB inflammatory signaling plays an important role in immune responses and survival of both normal and malignant cells. There are two traditional NF-κB activation pathways: the classical NF-κB activation pathway and the alternative NF-κB activation pathway. The classical pathway is triggered by the activation of the tripartite IKK complex composed of IKKα, IKKβ and IKKγ/NEMO, while the alternative pathway is mediated by IKKα-induced p100 processing [57,58,59]. Accumulating evidence suggests that NF-κB signaling in both PCa cells and immune/inflammatory cells plays an important role in the emergence and maintenance of CRPC. In PCa cells, NF-κB inflammatory signaling is constitutively activated to facilitate PCa cell AI growth and survival by up-regulating the expression of survival genes, such as cytokines (CXCL13, the receptor activator of nuclear factor-kappaB ligand (RANKL), etc.) and stem cell transcription factors (TWIST2, SOX2, OCT4 and NANOG, etc.). Meanwhile, abnormal activation of NF-κB inflammatory signaling in tumor-associated stromal cells, such as myeloid-derived suppressor cells (MDSCs) and tumor-associated macrophages (TAMs), up-regulates the production of pro-inflammatory cytokines, chemokines and growth factors, which facilitates the formation of immunosuppressive TME and promotes the growth and development of CRPC [59,60]. It was reported that ursolic acid, a pentacyclic triterpenoid, inhibits IκB kinase activation and IκBα phosphorylation in PCa cells. Treatment with ursolic acid inhibits TNFα-induced NF-κB activation and the STAT3 signal pathway, resulting in decreased PCa cell proliferation in vitro and xenograft tumor growth in animal models [32]. Apigenin, a common plant flavonoid, can inhibit IKKα activation, IκBα phosphorylation and degradation. Treatment with apigenin leads to down-regulation of NF-κB-regulating genes, such as Bcl-2, cyclooxygenase-2 (COX-2) and matrix metalloproteinase 9 (MMP-9), and sensitizes hormone-refractory PCa cells to TNFα-induced apoptosis [33]. It was also reported that α-tomatine suppresses NF-κB signaling in PCa cells, resulting in down-regulation of NF-κB-dependent anti-apoptotic genes, such as Bcl-2, Bcl-xL and survivin (Figure 1 and Figure 2, Table 1) [34].

### 2.2. TANK-Binding Kinase 1 (TBK1)

The TBK1, a non-canonical IKK family member, plays a pivotal role in antiviral innate immunity. TBK1 is a critical component of antiviral responses through phosphorylation and activation of interferon regulatory factor 3 (IRF3), IRF7, and STAT1. Lipopolysaccharide (LPS) treatment induces TBK1/IKKi-dependent phosphorylation and activation of IRF3 in macrophages [61,62]. It was reported that TBK1 regulates PCa dormancy and chemotherapeutic resistance in PC3 and C4-2B models. TBK1 can interact with mTOR and reduce its activity; knockdown of TBK1 decreases drug resistance of PCa cells [63,64]. Currently, there are several TBK1 inhibitors available for laboratory use and preclinical studies. TBK1 inhibitor BX795 inhibits tumor growth through inducing mitotic phase arrest and cell apoptosis in a pre-clinical model of oral squamous cell carcinoma (OSCC) [65]. The combined treatment of BX795 and MEK inhibitor AZD6244 enhances apoptosis in AZD6244-resistant and NRAS mutated melanoma [66]. We reported that TBK1 and IKKi, another non-canonical IKK family member, can functionally compensate for each other. Individual knockdown of either IKKi or TBK1 have minor effects on cell survival while simultaneous knockdown of both TBK1 and IKKi significantly inhibited cell proliferation, suggesting that simultaneously targeting both TBK1 and IKKi is necessary for the efficient shutdown of cancer cell growth [62]. We developed TBK1/IKKi dual inhibitors, SR8185, 200A, and 200B that show potent anti-tumor activity in human breast, prostate, and oral cancer cells. These inhibitors function partially through suppression of TBK1/IKKi-mediated AKT phosphorylation and vascular endothelial-derived growth factor (VEGF) expression [62].

### 2.3. Protein Kinase A (PKA)

The PKA, composed of two regulatory subunits (R) and two catalytic subunits (C), is an enzyme whose activity is dependent on cellular cyclic AMP (cAMP) levels. The PKA is inactive when the two R subunits dimerize and bind to the C subunits. However, when cAMP binds to the R subunits, PKA undergoes a conformation change that leads to the release and free of active C subunit, and the phosphorylation of its substrates [35,67]. In PCa, PKA can synergize low levels of androgen during ADT to enhance androgen signaling and to promote the emergence of CRPC. PKA phosphorylates heat shock protein (HSP90), which binds to the unliganded AR in cytoplasm, leading to the release of AR from HSP90. The freed AR binds to HSP27 and translocates to the nucleus, resulting in up-regulation of AR targeting genes in androgen depleted environment. Treatment with PKA inhibitor H89 potently inhibits AR nuclear translocation [35]. It was also reported that PKA activity is regulated by PKA inhibitor proteins (PKIs) in PCa. The PKI family member PKIα binds to PKA that blocks PKA activity and disrupts PKA-mediated signaling events. PKIα acts as a molecular switch, diverting GPCR-Gαs-cAMP signaling toward EPAC-RAP1 and MAPK activation that promotes PCa growth, progression, and metastasis (Figure 1 and Figure 2, Table 1) [68].

### 2.4. PIM Kinase

PIM1 kinase, a serine/threonine kinase, belongs to the PIM family. PIM1 is a proto-oncogene and is associated with the development and progression of various human cancers, including PCa [69]. Two PIM1 kinase isoforms, PIM-1S and PIM-1L, are up-regulated in PCa, which promote PCa progression via modulating AR stability and transcriptional activity. PIM-1S phosphorylates AR at Ser213 that leads to recruitment of ubiquitin E3 ligase mdm2 and the degradation of AR, while PIM-1L phosphorylates AR at Thr-850 that results in recruitment of ubiquitin E3 ligase RING finger protein 6 (RNF6) and stabilization of AR [70]. Treatment with PIM1 inhibitors compound 9 and its pyrrolo[2,3-a]carbazole derivatives suppresses the proliferation of PCa cells [36]. It was observed that PIM kinases, PIM1 and PIM3, promote PCa cell migration and growth through phosphorylating CXCR4 chemokine receptor. Treatment with PIM kinases inhibitor DHPCC-9 suppresses PCa growth and metastasis (Figure 1 and Figure 2, Table 1) [37].

### 2.5. Tumor Progression Locus 2 (Tpl2)

Tpl2 is a serine/threonine protein kinase and acts as an essential modulator of immune responses. Tpl2 has been associated with tumor progression, metastasis, and therapy resistance [71]. The expression of Tpl2 in metastatic PCa is much higher than that in local primary PCa, and Tpl2 is associated with the emergence and maintenance of CRPC [6,72]. Tpl2 activates CXCL12/CXCR4 and focal adhesion kinase (FAK)/AKT signal pathway to promote PCa AI survival, metastasis, and chemoresistance. Tpl2 could be a promising therapeutic target for CRPC and metastatic CRPC (mCRPC) [6]. Several Tpl2 inhibitors, such as 1,7-naphthyridine-3-carbonitriles and quinoline-3-carbonitriles, were developed for the treatment of autoimmune diseases [73]. Interferon-α (IFNα) is identified as a natural inhibitor of Tpl2 in bladder cancer. IFN-α inhibits Tpl2 phosphorylation and suppresses COX-2 expression [74].

### 2.6. NEK6

NEK6, a mitotic-related serine/threonine kinase, is activated in cell mitosis and is required for mitotic spindle formation and cytokinesis [75]. Abnormal expression of NEK6 has been reported in human cancers, including hepatic cancer, breast cancer and PCa [3,76]. NEK6 interacts with and phosphorylates STAT3, leading to activation of STAT3-mediated transcription and transformation of JB6 Cl41 mouse epidermal cells [3]. In PCa, NEK6 phosphorylates transcription factor FOXJ2 and induces PCa AI growth. Knockdown of NEK6 restores the sensitivity of CRPC cells to ADT in PCa xenograft models [76], suggesting that combined NEK6 inhibition with ADT would be an effective therapeutic strategy for CRPC.

### 2.7. Glycogen-Synthase-Kinase 3β (GSK-3β)

GSK-3 is a serine/threonine kinase, which includes isoform GSK3α and GSK3β. GSK-3 participates in many important cellular events through phosphorylating its substrates, such as CCAAT/enhancer-binding protein (C/EBPα) and beta-catenin. GSK-3 is associated with human cancer development and progression [77]. Inhibition of GSK3β induces the AR nuclear export, resulting in growth inhibition of AR-positive CRPC cells [78]. There is a functional link between GSK-3, β-catenin and AR-V7 in CRPC cells. Inhibition of GSK-3 by inhibitor LY-2090314 activates β-catenin signaling, which sequentially suppresses AR-V7 transcription activity. Therefore, AR-V7-positive PCa cells are more vulnerable to LY-2090314 treatment than AR-V7-negative PCa cells [41]. In addition, GSK3β activity can be regulated by aspartyl (asparaginyl) β hydrolase (ASPH). Knockdown of ASPH increases the phosphorylation of GSK3β, suggesting that ASPH may interfere with the interaction of GSK3β with its upstream kinases (Figure 1 and Figure 2, Table 1) [79].

### 2.8. T-LAK Cell-Originated Protein Kinase (TOPK)

TOPK is characterized as a mitogen-activated serine/threonine protein kinase. TOPK is not expressed or is expressed at a very low level in most normal adult tissues; however, it is highly expressed in various cancers, including PCa. High expression of TOPK in PCa is associated with advanced stages, aggressiveness, and metastasis [26,80]. TOPK drives PCa cell AI growth via up-regulation of AR splice variant (AR-V7). Treatment with TOPK inhibitor OTS-514 suppresses AR transactivation and stability [26].

### 2.9. Cyclin-Dependent Kinases (CDKs)

The CDKs family are a group of different kinases that are involved in regulation of cell cycle, transcription, metabolism, and many other cellular events. To be active, CDKs need bind to a cyclin protein, and different combinations of specific CDKs and cyclins mark different parts of the cell cycle [81]. CDK mutations and abnormal activation have been found in many cancers, including PCa. CDK4/6 together with cyclin D1 regulates the phosphorylation of retinoblastoma tumor suppressor (Rb), which determines S phase entrance. Treatment with 3, 9-dihydroxy-2-prenylcoumestan (pso), a furanocoumarin and a CDK4 inhibitor, induces G0/G1 cell cycle arrest and growth inhibition of CRPC cells [29]. CDK12 plays an important role in transcriptional regulation, RNA splicing, and DNA integration. CDK12 mutations/loss are associated with genomic instability and have been observed in many human cancers, including PCa [82,83]. PCa with CDK12 mutations progresses rapidly to metastasis and castration resistance [83]. Abnormal CDK7 activation has been found in many cancers, including PCa, and CDK7 inhibitors have potent antitumor activity [30,84]. CDK7 can phosphorylate AR coactivator MED1 in CRPC cells. CDK7 inhibition, either through genetic silence or its inhibitor such as THZ1, interferes with the recruitment of MED1 to chromatin that suppresses the expression of AR regulated genes, resulting in inhibition of CRPC development [30,31]. CDK9 is a key component of P-TEFb transcription elongation complex. CDK9 has also been identified as a cofactor for AR, MYC and other oncogenic transcription factors [28,85]. A selective CDK9 inhibitor, KB-0742, shows potent anti-tumor activity in CRPC models. Treatment with KB-0742 rapidly down-regulates nascent transcription and AR-driven oncogenic programs in CRPC cells (Figure 1 and Figure 2, Table 1) [28].

### 2.10. Bromodomain (BRD)-Containing Kinases (BETs)

BETs are master regulators of global transcription elongation. BETs have been identified as attractive therapeutic targets for anti-viral infection, inflammation, and cancer [86]. BETs promote CRPC development by regulating AR-, ERG-, and c-Myc-mediated transcription [87]. BRD4, a bromodomain and extra terminal domain (BET) protein, interacts with N-terminal domain of AR to regulate AR signaling [88]. BRD4 also binds to super-enhancers (SEs), which drives high expression of oncogenes in tumor cells. BETs inhibitor JQ1 shows potent anticancer activity in PCa cells through down-regulation of SE-associated genes [16]. MZ1, a BRD4-selective degrader, inhibits metastasis of CRPC cells [89]. In addition, BRD4 is a H3K27ac reader and involves in CHPT1 super enhancer (SE) activity and CHPT1 expression in enzalutamide (Enz)-resistant CRPC cells [90]. BRD4 is also reported as a vital component of BRD4-KDM5C-PTEN oncogenic pathway in CRPC development. BRD4 up-regulates lysine-specific histone demethylase 5C (KDM5C) expression, which represses phosphatase and tensin homolog (PTEN) gene [91]. BRD4 is also a targeted gene of microRNA-200a (miR-200a), miR-200a suppresses CRPC development via interfering with the BRD4-mediated AR signaling [92]. Treatment with BRD4 inhibitors (MA4-022-1 compound and analogues) inhibits PCa cell proliferation and migration [17]. Two benzo[d]isoxazole derivatives, 6i (Y06036) and 7m (Y06137), function as potent BRD4 inhibitors, which bind to BRD4 and suppress the expression of MYC and AR regulated genes [19]. (R)-12, a benzoxazinone-containing 3,5-dimethylisoxazole derivative, is a newly developed BET inhibitor, which binds to BRD4 and blocks the interaction between bromodomain and acetyl lysine. Treatment with (R)-12 suppresses ERG, Myc and AR signaling, and inhibits CRPC cell proliferation, colony formation, and tumor growth [18]. Treatment with BET inhibitor ABBV-075 disrupts BRD4 recruitment and down-regulates AR target genes [20]. 7d, one of the several diphenylamine derivatives developed for the inhibition of BET activity in CRPC, exhibits favorable metabolic stability and might be a promising agent for the treatment of clinical CRPC (Figure 2 and Table 1) [21].

### 2.11. Aurora Kinase A (AURKA)

AURKA is a cell cycle-regulated kinase that is involved in microtubule formation and/or stabilization at the spindle pole during chromosome segregation. AURKA is regulated by androgen, and is highly expressed in CRPC samples with amplification and/or high expression of AR. Treatment with AURKA specific inhibitor alisertib (MLN8237) significantly inhibits the growth of AR-overexpressing CRPC cells [7]. AURKA regulates androgen receptor variants (AR-Vs) expression, knockdown of AURKA leads to decreased synthesis of AR-V transcripts, such as AR-V7, but not the full-length AR. Treatment with AURKA inhibitor S1451 significantly decreases AR-V7 expression levels and markedly reduces AR-V-driven proliferation and survival of CRPC cells [25]. It has also been reported that AURKA phosphorylates Y-box binding protein-1 (YBX1), resulting in stabilization and nuclear translocation of YBX1 in CRPC cells. In turn, YBX1 also stabilizes AURKA, and therefore forming an AURKA-YBX1 synergistic loop that promotes PCa epithelial to mesenchymal transition (EMT), progression and chemoresistance [93]. SPOP, an adapter protein for E3 ubiquitin ligase, is a direct substrate of AURKA. AURKA induces SPOP degradation via phosphorylation of SPOP, leading to stabilization of AR, AR-V7, and c-Myc. Treatment with AURKA inhibitor alisertib (MLN8237) sensitizes CRPC cells to enzalutamide (Figure 1 and Figure 2, Table 1) [94].

### 2.12. AMP-Activated Protein Kinase (AMPK)

AMPK is a conserved serine/threonine protein kinase and is regarded as a metabolic sensor in mammalian cells. Forkhead box protein M1 (FOXM1) drives CRPC docetaxel resistance via induction of AMPK/mTOR-mediated autophagy [95]. Prostate Leucine Zipper (PrLZ), an important gene for CRPC development, promotes PCa docetaxel resistance through suppression of LKB1/AMPK-mediated autophagy [96].

### 2.13. Mitogen-Activated Protein Kinase (MAPK) and Jun Kinase (JNK)

Abnormal MAPK activation promotes CRPC development, resulting in early relapse and short disease-free survival [97]. A p38 MAPK and heat shock protein 27 (Hsp27) driven signaling axis has been identified as an important regulator of AR activity in hormone-sensitive PCa cells. Inhibition of p38 MAPK suppresses Hsp27 and the hypoxia-mediated AR activation and CRPC development in PCa mouse models [98]. ADT induces a compensatory activation of MAPK or JNK signaling, combination of JNK inhibitor AS602801 with enzalutamide synergistically inhibits PCa cell proliferation, invasion, and migration in vitro and xenograft tumor development in mouse models [99].

### 2.14. Phosphatidylinositol-3-Kinase (PI3K)/AKT

PI3K/AKT pathway plays an important role in cell metabolism, growth, proliferation, and survival. Abnormal activation of PI3K/AKT pathway is closely associated with CRPC development [100]. The combined treatment of enzalutamide with AKT inhibitor (AZD5363) significantly slows down enzalutamide-resistant PCa progression in mouse models [101]. Targeting both the PI3K/AKT pathway and androgen receptor axis significantly delays CRPC development in animal models [102]. The combined treatment of GRT (an aspalathin-rich green rooibos extract), Bcl-2 inhibitor ABT-737, PI3K inhibitor LY294002, and AKT inhibitor GSK 690693 exhibits potent inhibitory effect on CRPC development [103]. Treatment with PI3K specific inhibitor wortmannin and AKT inhibitor MK2206 blocks PI3K/AKT signaling and inhibits PCa cell invasion and colony formation [104]. In addition, the combination of Bcl-2 inhibitor ABT-737 with AKT inhibitor erufosine also shows a very potent anticancer effect on CRPC cells (Figure 1 and Figure 2, Table 1) [105].

### 2.15. Polo-Like Kinase 1 (PLK1)

PLK1 belongs to polo-like kinases (PLKs) family, which contains four serine/threonine protein kinases that are critical regulators in cell cycle progression, mitosis, cytokinesis, and DNA damage responses [106]. PLK1 is overexpressed in PCa and is associated with higher grade tumors. PLK1 signaling is one of the most activated pathways after ADT. PLK1 activation promotes CRPC development through PI3K–AKT–mTOR pathway as well as AR and Wnt/β-catenin signaling. PLK1 inhibition sensitizes PCa cells to androgen signaling inhibitors [107,108]. Targeting PLK1 with specific inhibitor BI2536 enhances the effect of PARP inhibitor on CRPC cells [13]. As PLK1 inhibition leads to β-catenin signaling activation c-MYC overexpression, PLK1 inhibitor GSK461364A shows a strong synergistic inhibitory effect on CRPC cells when combined with BRD4 inhibitor JQ1, which inhibits c-MYC expression and AR signaling [14]. Treatment with BETs and PLK1 dual inhibitor WNY0824 significantly suppresses AR-positive CRPC cell proliferation in vitro and xenograft tumor growth in animal models (Figure 1 and Figure 2, Table 1) [15].

### 2.16. LIM-Domain Kinase-2 (LIMK2)

LIMK2, a serine/threonine and tyrosine kinase, contains two LIM motifs, a PDZ and kinase domain. The expression of LIMK2 is very high in PCa, and is further increased after ADT. LIMK2 promotes CRPC development through phosphorylation and stabilization of TWIST1 [10]. In addition, LIMK2 phosphorylates and degrades SPOP, which results in stabilization of AR, AR-V7 and c-MYC in CRPC cells [109]. LIMK2 also phosphorylates and degrades PTEN [110]. LIMK2 allosteric inhibitor (LI) shows high synergistic effect with docetaxel on CRPC cells [10,109].

## 3. Tyrosine Kinase and Their Therapeutic Implications

Tyrosine kinases phosphorylate tyrosine amino acid residues, which function primarily as growth factor receptors or/and in downstream signaling of growth factors. Tyrosine kinases include receptor tyrosine kinases (RTKs) and non-receptor tyrosine kinases (NRTKs). Abnormal activation of tyrosine kinases is associated with the development of various cancers, including PCa [111,112].

### 3.1. The Receptor Tyrosine Kinases (RTKs)

The epidermal growth factor receptor (EGFR, also known as HER1) is a receptor tyrosine kinase and is a member of ErbB family [112]. Abnormal EGFR signaling is associated with PCa pathogenesis, progression, and CRPC development. It was reported that the nuclear localization of EGFR can be regulated by Annexin A1, and the increased nuclear EGFR promotes CRPC development. Cyclosporin H can interrupt nuclear EGFR and its downstream signaling, which consequently suppresses PCa growth [113]. EGFR induces CRPC docetaxel resistance via regulating Akt-ABCB1 signaling. Treatment with EGFR inhibitor gefitinib significantly suppresses proliferation of docetaxel-resistant PCa cells in vitro and xenograft tumor growth in animal models (Figure 2 and Table 1) [40].

Human epidermal growth factor receptor 2 (HER2) is another member of ErbB family, which is well known to be associated with breast cancer. HER2 is also involved in AR activation and PCa progression [41,56,114]. Lapatinib, a dual EGFR and HER2 inhibitor, has been applied in a phase II trial and shown potent initial antitumor activity in CRPC patients [56]; however, development of lapatinib resistance limits its clinical application. The FDA-approved ErbB inhibitor dacomitinib, at equimolar concentrations as lapatinib, can decrease HER2 protein stability and prevent EGFR/HER2 heterodimerization that lead to interrupting downstream signaling and increasing CRPC cell apoptosis [41]. It has been reported that both MET and VEGFR signal pathways play an important role in CRPC [115]. Dual inhibition of MET and VEGFR by cabozantinib (formerly XL184, a multi-targeted tyrosine kinase inhibitor) or the combined axitinib (VEGFR inhibitor) with crizotinib (MET inhibitor) shows strong synergistic inhibitory effect on CRPC (Figure 2 and Table 1) [115,116].

### 3.2. The Non-Receptor Tyrosine Kinases (NRTKs)

The rearranged during transfection (RET) is a tyrosine kinase and plays an important role in cell growth and differentiation. Abnormal RET expression is reported in various cancers, including PCa. RET expression is increased in neuroendocrine PCa (NEPC), and is associated with neuroendocrine transcription factors, such as POU3F2, SOX2, ONECUT2, and ASCL1. Treatment with RET inhibitor AD80 induces cell death and suppresses tumor development in NEPC xenograft models [42]. The Lck/yes-related protein tyrosine kinase (Lyn) belongs to the Src kinase family (SFK) and plays a pivotal role in the pathogenesis of various tumors. The expression of Lyn tyrosine kinase is increased in human CRPC as compared with hormone naive PCa or normal prostate tissue. Overexpression of Lyn enhances AR transcriptional activity and accelerates CRPC progression. Targeting Lyn kinase induces AR dissociation from its molecular chaperone Hsp90, leading to AR ubiquitination and degradation [117]. Ack1 tyrosine kinase phosphorylates AR at Tyr-267, treatment with Ack1 inhibitor AIM-100 suppresses AR-Tyr-267 phosphorylation and Ack1 mediated ATM (ataxia telangiectasia mutated) expression as well as the growth of radioresistant CRPC (Figure 2 and Table 1) [118].

The Janus family kinases (Jaks), Jak1, Jak2, Jak3, and Tyk2, form one subgroup of the NRTKs. Jaks are involved in cell growth, survival, development, and differentiation. The activation of Jaks by IFNs induces phosphorylation of transcription factor Stat1 at Y701, leading to Stat1 dimerization and translocation to nucleus. Another protein tyrosine kinase Pyk2 also plays a critical role in the Jak-mediated MAPK and Stat1 activation [119]. JAK2 can mediate both tyrosine phosphorylation and serine phosphorylation [120]. JAK2 is overexpressed in PCa cells. Jak2 inhibitor AZD1480 inhibits Stat5a/b phosphorylation, dimerization, nuclear translocation, DNA binding and transcriptional activity in PCa cells. Treatment of Jak2 inhibitor AZD1480 suppresses PCa xenograft tumor development, and prolongs survival time of tumor-bearing mice as compared with vehicle or docetaxel-treated groups [121]. In addition, it was reported that β-elemonic acid (β-EA), a natural triterpene, inhibits growth and triggers apoptosis of human CRPC cells through the suppression of JAK2/STAT3/MCL-1 and NF-ĸB signaling pathways (Figure 2 and Table 1) [39].

## 4. Lipid Kinases and Their Therapeutic Implications

Lipid kinases phosphorylate lipids in the cell, both on the plasma membrane and on the membranes of the organelles. The addition of phosphate groups can change the reactivity and localization of the lipid, leading to signal transmission.

### 4.1. Phosphatidylinositol 4-Phosphate 5-Kinase Type 1α (PIP5K1α)

PIP5K1α belongs to the family of PI-4-phosphate 5 kinases (PIP5Ks) and contacts the substrate PI4P via two major regions of an arginine/lysine-rich region (the β8-α4ϲ loop) and the activation loop (Protein Data Bank (PDB: 4TZ7) residues 378–415, a structure of PIP5K1α) [122]. PIP5K1α is upstream of PI3K since PIP5K1α produces a substrate of PI3K, PtdIns-4,5-P2 (PIP2), which is required for the activation of PI3K/AKT pathway. The activation of PI3K/AKT signaling is observed in various cancers, including PCa [43]. PIP5K1α is highly expressed in advanced and metastatic PCa as compared with primary tumors, and PIP5K1α expression is positively correlated with AR expression. Overexpression of PIP5K1α increases AKT activity and the invasiveness of PCa cells. Knockdown of PIP5K1α or treatment with its inhibitor ISA-2011B significantly inhibits AKT activity, increases cancer cell apoptosis and suppresses CRPC growth in animal models (Figure 2 and Table 1) [43].

### 4.2. Sphingosine Kinases (SKs)

SKs are lipid kinases that catalyze the conversion of sphingosine to sphingosine-1-phosphate (S1P). On activation, SKs migrate from cytosol to plasma membrane where they transfer a γ phosphate from ATP or GTP to sphingosine. There are two SKs in mammalian cells, SK1 and SK2. SK1 is highly expressed in certain types of cancers, including PCa [123]. SK1 expression level and activity are associated with PCa progression, recurrence, and chemotherapy resistance [44,123,124]. AI PCa PC3 cells have much higher SK1 activity than AD PCa LNCaP cells, and SK1 involves in AI survival of PCa cells [123,125]. Treatment with SK1 inhibitor FTY720 (fingolimod) activates caspase-3 and induces apoptosis in DU145 cells. FTY720 treatment also sensitizes CRPC cells to radiotherapy. Furthermore, SK1 inhibition overcomes enzalutamide resistance and enhances enzalutamide efficacy in CRPC cells [44,45,46]. It was also reported that treatment with SK2 inhibitor ABC294640 reduces CRPC cell growth as well as the expression of c-Myc and AR. ABC294640 treatment also inhibits dihydroceramide desaturase (DEGS), resulting in the increase of dihydroceramides (Figure 2 and Table 1) [47].

## 5. Carbohydrate Kinases and Their Therapeutic Implications

### 5.1. Hexokinase

Hexokinase is the most common enzyme in glucose metabolism, which converts D-glucose to glucose-6-phosphate by transferring the gamma phosphate of an ATP to the C6 position. Elevated expression of hexokinase 2 (HK2) has been identified in several cancers [126]. In PCa, increased HK2 expression and activity are associated with CRPC development, especially PTEN- and TP53-deficiency-driven CRPC [48,126,127]. Under ADT, HK2 stability is regulated by IL13Rα1 in PCa cells. IL13Rα1 recruits ubiquitin protein ligase E3C to mediate ubiquitination and degradation of HK2, leading to glycolytic inhibition and PCa cell apoptosis [128]. Targeting HK2 with its inhibitor 2-deoxyglucose (2-DG) suppresses CRPC growth. When 2-DG is combined with chloroquine (CQ), which targets ULK1-dependent autophagy, the treatment leads to a near-complete tumor suppression and remarkably extend survival in PTEN- and TP53-deficiency-driven CRPC animal models (Figure 2 and Table 1) [48].

### 5.2. Phosphofructokinase (PFK)

PFK catalyzes the conversion of fructose-6-phosphate to fructose-1,6-bisphosphate and is a key enzyme in the regulation of glycolysis. PFK acts as an important mediator of metabolism in cancer cells to support growth and survival. The expression level of PFK is elevated in several cancers, such as breast cancer, PCa, and lung cancer; the expression of phosphofructokinase platelet (PFKP), an isoform of PFK, is associated with poor prognosis of breast cancer [129]. In CRPC, PFK is associated with PCa cell proliferation and its expression is regulated by KDM4B [130].

## 6. Other Kinases and Their Therapeutic Implications

In addition to the kinases that phosphorylate proteins, lipids, and carbohydrates, there are some kinases acting on nucleotides, such as nucleoside-phosphate kinases and nucleoside-diphosphate kinases. Other small molecules such as creatine, phosphoglycerate, riboflavin, dihydroxyacetone, shikimate can also be substrates of kinases.

### 6.1. Riboflavin Kinases (RFK)

RFK catalyzes the phosphorylation of riboflavin to create flavin mononucleotide (FMN), which is an important cofactor and a precursor to flavin adenine dinucleotide (FAD) as well as a redox cofactor used by many enzymes in metabolism. It has been reported that RFK involved in cisplatin resistance and cellular protection from oxidative stress in PC3 cells. Knockdown of RFK enhances the sensitivity of PC3 cells to cisplatin [131].

### 6.2. Thymidine Kinase

Thymidine kinase is one of the nucleoside kinases that is responsible for nucleoside phosphorylation. It phosphorylates thymidine to create thymidine monophosphate (dTMP). Thymidine kinase activity is closely correlated with cell cycle and has been used as a tumor marker in clinical chemistry [132]. Osteocalcin-thymidine kinase (OC-TK) is highly expressed in AI PCa cells as compared with AD PCa cells [133]. In addition, the concentrations of serum thymidine kinase-1 (TK-1) are correlated with PCa stages. Together with PSA, TK-1 can be used as a diagnostic marker for PCa [134].

## 7. Conclusions and Perspectives

ADT was first developed in 1941 by Dr. Charles Huggins, who won the Nobel Prize in Physiology and Medicine in 1966, and still remains the principal and backbone treatment for patients with advanced PCa, because the majority of patients respond to ADT with a mean remission time of 2–3 years, which is a big advantage for those patients with advanced PCa. Currently there is no better systemic therapy that could replace ADT for the treatment of advanced PCa. However, once it becomes castration-resistant, the PCa is lethal and incurable. Many kinase-mediated signaling pathways are abnormally activated and involved in the pathogenesis of CRPC. And, therefore, kinases have been regarded as one of the most important and promising targets for the treatment of CRPC. Small molecule inhibitors exhibit good penetration and accessibility as compared with other large molecules; many efforts have been made to develop small molecule inhibitors that target kinases mutated or abnormally activated in CRPC (Figure 1 and Figure 2). Many of these small molecule inhibitors are still at the early stages in preclinical studies, some are in clinical trials (Table 1 and Table 2).

Multiple kinases or pathways can be activated simultaneously in one single tumor cell, and different tumor cells in the same tumor have different kinase or pathway activation. Additionally, one kinase or pathway can be compensated for by other kinases or pathways once being targeted. Although we appreciate that most of these available kinase inhibitors are designed to target one specific kinase, which can avoid side (off-target) effects caused by nonspecific inhibition of other targets important for normal cells, targeting one single kinase or pathway may not be efficient or no effect at all for the suppression of tumor development. For instance, both MET and VEGFR signaling play an important role in CRPC development [115]; the inhibition of MET and VEGFR by a multi-targeted tyrosine kinase inhibitor cabozantinib shows much stronger anti-CRPC activity than either VEGFR inhibitor axitinib or MET inhibitor crizotinib used alone [115,116]. We reported that TBK1 and IKKi can functionally compensate for each other. Knockdown of either IKKi or TBK1 has a minor effect on cell survival while simultaneous knockdown of both TBK1 and IKKi significantly inhibits cell proliferation [62]. TBK1/IKKi dual inhibitors show potent anti-tumor activity in human breast, prostate, and oral cancer cells [62].

Currently, most kinase inhibitors are developed to target the kinases in cancer cells. tumor is composed of not only cancer cells but also many types of stromal cells, such as tumor-associated macrophages (TAMs), myeloid-derived suppressor cells (MDSCs), T cells, B cells, and NK cells [12,135]. These stromal cells, particularly infiltrated immune/inflammatory cells, either contribute to or are affected by the immunosuppressive tumor microenvironment (TME) [136]. The abnormal signaling mediated by kinase activation/suppression in immune/inflammatory cells in TME would be potential good targets. Therefore, development of therapeutic strategies, particularly small molecule inhibitors/activators that could not only inhibit abnormal kinase activation in tumor cells to suppress tumor cell proliferation, but also modify the kinases activity in immune/inflammatory cells to tilt the balance of TME from immune suppression to effective immune surveillance, or/and to restore the tumor-killing function of “exhausted” CD8+ T cells, would be highly appreciated.

## Data Availability

Not applicable.
